# An EKF-Based Fixed-Point Iterative Filter for Nonlinear Systems

**DOI:** 10.3390/s19081893

**Published:** 2019-04-21

**Authors:** Xiaoliang Feng, Yuxin Feng, Chenglin Wen

**Affiliations:** 1 College of Electrical Engineering, Henan University of Technology, Zhengzhou 450001, China; fyxxq0126@163.com (Y.F.); wencl@hdu.edu.cn (C.W.); 2 School of Automatic, Hangzhou Dianzi University, Hangzhou 310018, China

**Keywords:** fixed-point filter, extended Kalman filter, nested iterative method, Steffensen’s iterative method, convergence condition

## Abstract

In this paper, a fixed-point iterative filter developed from the classical extended Kalman filter (EKF) was proposed for general nonlinear systems. As a nonlinear filter developed from EKF, the state estimate was obtained by applying the Kalman filter to the linearized system by discarding the higher-order Taylor series items of the original nonlinear system. In order to reduce the influence of the discarded higher-order Taylor series items and improve the filtering accuracy of the obtained state estimate of the steady-state EKF, a fixed-point function was solved though a nested iterative method, which resulted in a fixed-point iterative filter. The convergence of the fixed-point function is also discussed, which provided the existing conditions of the fixed-point iterative filter. Then, Steffensen’s iterative method is presented to accelerate the solution of the fixed-point function. The final simulation is provided to illustrate the feasibility and the effectiveness of the proposed nonlinear filtering method.

## 1. Introduction

Interesting but unavailable signal variables can be estimated using a proper filter. In recent decades, filter design for various theoretical and applied systems has been a popular research topic in the fields of automatic control, target tracking, fault diagnosis, etc. [1,2,3,4,5].

The Kalman filter is designed for linear systems with noise-satisfying Gaussian distribution, and is an optimal filter due to the minimum mean square error (MMSE) [6]. The Kalman filter provides an estimate and prediction of the system state in real time. However, the classical Kalman filter must be applied to linear systems. For the filtering problem of nonlinear systems, a large number of effective filters have been developed on the basis of the classical Kalman filter.

The filters for nonlinear systems are mainly designed according to two principles. The first kind of nonlinear filter is designed by linearizing the system function, such as the celebrated extended Kalman filter (EKF) [7]. The second kind of nonlinear filter is designed on the basis of approximating the state statistics, such as the Unscented Kalman filter (UKF) [8,9,10], and the Cubature Kalman filter (CKF) [11,12,13]. Various nonlinear filters are designed in different ways to predict and update nonlinear system state estimates. In the state estimate prediction stage, according to EKF, the process function is linearized by discarding the second or higher order Taylor series items at the state estimate of the previous time instant. Then, the Kalman filter is utilized to predict the state estimate in terms of the linearized process function. In the state estimate updating stage, the measurement function is linearized by discarding the higher order items of its Taylor series at the predicted state estimate of the current time instant. Then, the Kalman filter is utilized to update the state estimate in terms of the linearized measurement function [14,15]. Although the EKF can estimate the state of a nonlinear system, the estimation accuracy of the EKF is always limited, due to the discarded second and higher order Taylor series terms of the process function and the measurement function in their linearization processes. In this context, a few nonlinear filters have been developed from the EKF. Based on the orthogonal principle, a fading factor is introduced to adjust the prediction error variance and to improve the robustness of the system modeling error and the filtering accuracy. The so-called strong tracking filter is proposed in [16,17], and developed in [18,19]. The multiple model method introduced in [20] simultaneously considers multiple possible state estimates, then improves the final state estimation accuracy through distributed extended Kalman filtering fusion. However, the discarded higher-order terms of the Taylor series in the linearization processes still inevitably affect the estimation accuracy of the nonlinear filters developed from the EKF.

The fixed-point theory is an effective numerical solution method for nonlinear equations. Therefore, the fixed-point theory is also utilized to design linear filters. In [21], the fixed-point theory is used for the identification of Wiener systems, which includes an infinite impulse response (IIR) system and a nonlinear static function. In [22,23], the maximum correntropy Kalman filter is designed by solving a fixed-point equation. The fixed-point iteration Gaussian sum filtering estimator with unknown time-varying non-Gaussian measurement noise is proposed in [24]. A characteristic function filter is developed in [25] for a class of non-Gaussian nonlinear dynamical systems with the linear measurement model.

Motivated by the discussion above, in this paper, a novel fixed-point nonlinear filtering method is studied to update the state estimate obtained by the EKF. Firstly, taking advantage of the fixed-point theory, a fixed-point function is constructed to solve the filtering problem. Since the state estimate and the filter gain in the fixed-point function are both unknown, a nested iterative method is presented to solve the fixed-point function and obtain the fixed-point filter. The convergence and the existing of the fixed-point filter are also discussed. Steffensen’s iterative method is presented to accelerate the solution of the fixed-point iterative function.

The main contributions of this paper are threefold. (1) A fixed-point function was utilized to reduce the influence of discarded second- and higher-order Taylor series terms of nonlinear systems and to improve the filtering accuracy of the steady-state EKF. (2) Two kinds of fixed-point function solution methods were used and resulted in two fixed-point iterative filters. (3) The convergence of the fixed-point function and the existing conditions of the fixed-point iterative filter are also provided. 

The remainder of this article is organized as follows. In Section 2, the general discrete time nonlinear system is formulated and the motivation of this paper is analyzed. In Section 3, a fixed-point function is provided to update the state estimate obtained by the EKF. The fixed-point filter is designed using two iterative solution methods, which iteratively update the state estimates and the filter gains. The convergence and existing conditions of the fixed-point filter are also discussed. The final illustrated simulation is provided in Section 4. Section 5 concludes the paper.

## 2. Problem Formulation

Considering the following nonlinear discrete-time system:

(1) x(k+ 1)=f(x(k),k)+w(k+ 1,k)

(2) y(k+ 1)=h(x(k+ 1),k)+v(k+ 1)

where x(k) is the system state at the discrete time instant k, f(*) is the nonlinear state evolution function and h(*) represents the nonlinear measurement function. The process noise w(k+1,k) and the measurement noise v(k+1) satisfy *w*(*k*+1,*k*) ~ N(0,*Q*(*k*+1,*k*)) and *v*(*k*+1) ~ N(0,*R*(*k*)).

**Remark 1.**  *The existing nonlinear filtering methods designed for the system above mainly include two types: (1) EKF-based nonlinear filtering methods, in which the nonlinear system model is always approximated by an appropriate linear function; and (2) sample point-based nonlinear filtering methods, in which the statistics of the state estimate are usually approximated by the statistical result of the sample points, for instance. However, it is unavoidable that certain errors exist in the above two kinds of approximations and result in estimation errors of the above two kinds of nonlinear filters. In order to improve the nonlinear filtering accuracy of the EKF-based nonlinear filtering methods, taking advantage of fixed-point theory, an EKF-based fixed-point iterative filter is proposed in this paper.*

## 3. EKF-Based Fixed-Point Filter

In this section, for the filtering problem of the general nonlinear system described in Equations (1) and (2), the classical EKF will firstly be introduced. Then, a fixed-point function will be provided to update the obtained state estimate by solving the fixed-point function. In Section 3.3, a nested iterative method is derived to solve the fixed-point filter. The convergence and existing condition of the fixed-point filter will be discussed in Section 3.4. Then, Steffensen’s iterative method will be presented to accelerate the solution of the fixed-point function in Section 3.5.

### 3.1. Classic Extended Kalman Filter

In the EKF-based nonlinear filtering methods for the system described in Equations (1) and (2), the nonlinear system model is always represented by the Taylor series at a working point and linearized by discarding the second and higher order terms. Then, the well-known Kalman filter is used to estimate the state of the linearized system. The classical EKF can be summarized as follows.

**Assumption 1.**  *The estimate and the estimation error variance of the state at the time instant *k*have been obtained and are denoted as*$\hat{x}(k|k),P(k|k)$*, then the state at the time instant*k+1 will be estimated as follows.

*(1) Time Updating*

*Taking the state estimate as the working point, the Taylor series expression of the nonlinear process function is*

(3) $$\begin{array}{l} x(k+1)=f(x(k),k)+w(k+1,k)\\ \;=f(\hat{x}(k|k),k)+{\frac{\partial f(x(k),k)}{\partial x(k)}|}_{x(k)=\hat{x}(k|k)}\left(x(k)-\hat{x}(k|k)\right)+O\left({\left(x(k)-\hat{x}(k|k)\right)}^{2}\right)+w(k+1,k)\\ \;\approx f(\hat{x}(k|k),k)+{\frac{\partial f(x(k),k)}{\partial x(k)}|}_{x(k)=\hat{x}(k|k)}\left(x(k)-\hat{x}(k|k)\right)+w(k+1,k) \end{array}$$

*Applying the Kalman filter to the linearized system shown as the second approximate formula above, the state prediction at the time instant*k+1*and the prediction error variance can be obtained by*

(4) $$\left\{ \begin{array}{l} \hat{x}(k+1|k)=f(\hat{x}(k|k),k),\\ P(k+1|k)=F(k+1,k)P(k|k)F^{T}(k+1,k)+Q(k+1,k), \end{array}\right.$$

*where*$F(k+1,k)={\frac{\partial f(x(k),k)}{\partial x(k)}|}_{x(k)=\hat{x}(k\;|\;k)}$*is the Jacobian of*f(x(k),k)*at the working point*$\hat{x}(k|k)$.

*(2) Measurement Updating:*

*Taking the state prediction obtained above as the working point, the measurement function can be rewritten as*

(5) $$\begin{array}{l} y(k+1)=h(x(k+1),k+1)+v(k+1)\\ \;=h(\hat{x}(k+1|k),k+1)+{\frac{\partial h(x(k+1),k+1)}{\partial x(k+1)}|}_{x(k+1)=\hat{x}(k+1|k)}\left(x(k+1)-\hat{x}(k+1|k)\right)\\ \;+O\left({\left(x(k+1)-\hat{x}(k+1|k)\right)}^{2}\right)+v(k+1)\\ \;\approx h(\hat{x}(k+1|k),k+1)+{\frac{\partial h(x(k+1),k+1)}{\partial x(k+1)}|}_{x(k+1)=\hat{x}(k+1|k)}\left(x(k+1)-\hat{x}(k+1|k)\right)+v(k+1) \end{array}$$

*The state estimate and the estimate error variance at time instant* k+1 *can be derived as*

(6) $$\left\{ \begin{array}{l} \hat{x}(k+1|k+1)=\hat{x}(k+1|k)+K(k+1)\left(y(k+1)-h(\hat{x}(k+1|k))\right),\\ P(k+1|k+1)=P(k+1|k)-K(k+1)H(k+1)P(k+1|k), \end{array}\right.$$

*where* $H(k+1)={\frac{\partial h(x(k+1),k+1)}{\partial x(k+1)}|}_{x(k+1)=\hat{x}(k+1|k)}$ *is the Jacobian of* h(x(k+1),k+1) *at the working point* $\hat{x}(k+1|k)$ *and* K(k+1) *is the filter gain, which is represented as*

(7) $$K(k+1)=P(k+1|k)H^{T}(k+1){\left(H(k+1)P(k+1|k)H^{T}(k+1)+R(k+1)\right)}^{-1}$$

### 3.2. Numerical Updating Method Based on the Fixed-Point Theory

As mentioned above, in the EKF, $\hat{x}(k+1|k+1)$ is obtained to estimate the system state x(k+1) on the basis of the measurements {y(1),y(2),⋯,y(k+1)}. Due to the discarded second and higher order Taylor series items of the process function and the measurement function, not all of the information contained in the measurement could be utilized to obtain $\hat{x}(k+1|k+1)$. In order to update this state estimate and make more full use of the unused information in the measurement, taking advantage of fixed-point theory, the following fixed-point function is constructed for a steady-state filter

(8) $$\left\{ \begin{array}{l} \hat{\hat{x}}(k+1|k+1)=\hat{\hat{x}}(k+1|k+1)+K^{*}(k+1)[y(k+1)-{\hat{y}}^{*}(k+1)]\\ {\hat{y}}^{*}(k+1)=h(\hat{\hat{x}}(k+1|k+1),k+1)+E\{v(k+1)\} \end{array}\right.$$

where $\hat{\hat{x}}(k+1|k+1)$ is the state of the above fixed-point filter, $K^{*}(k+1)$ is the filter gain to be determined, and E{v(k+1)} is the mean of the measurement noise, which was found to be zero in this paper.

**Remark 2.**  *In (8), the*$\hat{\hat{x}}(k+1|k+1)$*will be estimated on the basis of the measurements*{y(1),y(2),⋯,y(k+1)}*, such that the solution*$\hat{\hat{x}}(k+1|k+1)$*is close to the actual state value at time instant*k+1. *This aims to solve*$\hat{\hat{x}}(k+1|k+1)$*such that*$y(k+1)=h(\hat{\hat{x}}(k+1|k+1),k+1)$*. In order to solve*$\hat{\hat{x}}(k+1|k+1)$*, such that*$y(k+1)=h(\hat{\hat{x}}(k+1|k+1),k+1)+v(k+1)$, *the fixed-point function method is used, and (8) is the structured fixed-point function. However, as the state estimate and the filter gain are both unknown, it is difficult to solve the fixed-point function (8) by the traditional fixed-point iterative solution method.*

### 3.3. A Nested Iterative Solution for the Fixed-Point Function

In the fixed-point function (8), two unknown items must be determined simultaneously—The unknown state of the above fixed-point filter, $\hat{\hat{x}}(k+1|k+1)$ and the filter gain to be determined $K^{*}(k+1)$. In this section, a fixed-point filter is presented by solving the above fixed-point function (8), according to the following nested iterative computation process.

In the first iteration, take the state estimate $\hat{x}(k+1|k+1)$ obtained by the EKF as the initial value on the right side of the fixed-point function. Denote the initial state of the first iteration as ${\hat{\hat{x}}}_{0}^{0}(k+1|k+1)=\hat{x}(k+1|k+1)$, where the subscript 0 means the first iteration, while the superscript 0 is the initial value of this iteration, which increases in this iteration. Then we have the following iterative solution process of the fixed-point function with the initial filter gain $K_{0}^{*}(k+1)=\;K(k+1)$.

(9) $$\left\{ \begin{array}{c} {\hat{\hat{x}}}_{0}^{1}(k+1|k+1)={\hat{\hat{x}}}_{0}^{0}(k+1|k+1)+K_{0}^{*}(k+1)\left(y(k+1)-h({\hat{\hat{x}}}_{0}^{0}(k+1|k+1),k+1)\right)\\ {\hat{\hat{x}}}_{0}^{2}(k+1|k+1)={\hat{\hat{x}}}_{0}^{1}(k+1|k+1)+K_{0}^{*}(k+1)\left(y(k+1)-h({\hat{\hat{x}}}_{0}^{1}(k+1|k+1),k+1)\right)\\ \vdots \\ {\hat{\hat{x}}}_{0}^{i_{0}}(k+1|k+1)={\hat{\hat{x}}}_{0}^{i_{0}-1}(k+1|k+1)+K_{0}^{*}(k+1)\left(y(k+1)-h({\hat{\hat{x}}}_{0}^{i_{0}-1}(k+1|k+1),k+1)\right) \end{array}\right.$$

If $\left|{\hat{\hat{x}}}_{0}^{i_{0}}(k+1|k+1)-{\hat{\hat{x}}}_{0}^{i_{0}-1}(k+1|k+1)\right|<\varepsilon$, take ${\hat{\hat{x}}}_{0}^{i_{0}}(k+1|k+1)$ as the state estimate in the first iterative process. 

Due to

(10) $$\hat{\hat{x}}(k+1|k+1)=\hat{x}(k+1|k+1)+K_{}^{*}(k+1)\left(y(k+1)-h(\hat{\hat{x}}(k+1|k+1),k+1)\right)$$

the filter gain can be updated in the first iteration by

(11) $$K_{1}^{*}(k+1)=\frac{{\hat{\hat{x}}}_{0}^{i_{0}}(k+1|k+1)-{\hat{\hat{x}}}_{0}^{i_{0}-1}(k+1|k+1)}{y(k+1)-h({\hat{\hat{x}}}_{0}^{i_{0}-1}(k+1|k+1),k+1)}$$

In the second iteration, the initial value in the fixed-point numerical solving process is ${\hat{\hat{x}}}_{1}^{0}(k+1|k+1)=\;{\hat{\hat{x}}}_{0}^{i_{0}}(k+1|k+1)$, then we have

(12) $$\left\{ \begin{array}{c} {\hat{\hat{x}}}_{1}^{1}(k+1|k+1)={\hat{\hat{x}}}_{1}^{0}(k+1|k+1)+K_{1}^{*}(k+1)(y(k+1)-h({\hat{\hat{x}}}_{1}^{0}(k+1|k+1),k+1))\\ {\hat{\hat{x}}}_{1}^{2}(k+1|k+1)={\hat{\hat{x}}}_{1}^{1}(k+1|k+1)+K_{1}^{*}(k+1)(y(k+1)-h({\hat{\hat{x}}}_{1}^{1}(k+1|k+1),k+1))\\ \vdots \\ {\hat{\hat{x}}}_{1}^{i_{1}}(k+1|k+1)={\hat{\hat{x}}}_{1}^{i_{1}-1}(k+1|k+1)+K_{1}^{*}(k+1)(y(k+1)-h({\hat{\hat{x}}}_{1}^{i_{1}-1}(k+1|k+1),k+1)) \end{array}\right.$$

Similarly, if $\left|{\hat{\hat{x}}}_{1}^{i_{1}}(k+1|k+1)-{\hat{\hat{x}}}_{1}^{i_{1}-1}(k+1|k+1)\right|<\varepsilon$, ${\hat{\hat{x}}}_{1}^{i_{1}}(k+1|k+1)$ is taken as the state estimate in the second iterative process, then the filter gain in the second iteration can be updated by

(13) $$K_{2}^{*}(k+1)=\frac{{\hat{\hat{x}}}_{1}^{i_{1}}(k+1|k+1)-{\hat{\hat{x}}}_{1}^{i_{1}-1}(k+1|k+1)}{y(k+1)-h({\hat{\hat{x}}}_{1}^{i_{1}}(k+1|k+1),k+1)}$$

For the *l*th iteration, ${\hat{\hat{x}}}_{l}^{0}(k+1|k+1)=\;{\hat{\hat{x}}}_{l-1}^{i_{l-1}}(k+1|k+1)$ is taken as the initial value to solve the fixed-point numerical and is iteratively updated by the following

(14) $$\left\{ \begin{array}{c} {\hat{\hat{x}}}_{l}^{1}(k+1|k+1)={\hat{\hat{x}}}_{l}^{0}(k+1|k+1)+K_{l}^{*}(k+1)(y(k+1)-h({\hat{\hat{x}}}_{l}^{0}(k+1|k+1),k+1))\\ {\hat{\hat{x}}}_{l}^{2}(k+1|k+1)={\hat{\hat{x}}}_{l}^{1}(k+1|k+1)+K_{l}^{*}(k+1)(y(k+1)-h({\hat{\hat{x}}}_{l}^{1}(k+1|k+1),k+1))\\ \vdots \\ {\hat{\hat{x}}}_{l}^{i_{l}}(k+1|k+1)={\hat{\hat{x}}}_{l}^{i_{l}-1}(k+1|k+1)+K_{l}^{*}(k+1)(y(k+1)-h({\hat{\hat{x}}}_{l}^{i_{l}-1}(k+1|k+1),k+1)) \end{array}\right.$$

Similarly, if $\left|{\hat{\hat{x}}}_{l}^{i_{l}}(k+1|k+1)-{\hat{\hat{x}}}_{l}^{i_{l}-1}(k+1|k+1)\right|<\varepsilon$, ${\hat{\hat{x}}}_{l}^{i_{l}}(k+1|k+1)$ is taken as the state estimate in the *l*th iterative process, then the filter gain in the *l*th iteration can be updated by

(15) $$K_{l+1}^{*}(k+1)=\frac{{\hat{\hat{x}}}_{l}^{i_{l}}(k+1|k+1)-{\hat{\hat{x}}}_{l}^{i_{l}-1}(k+1|k+1)}{y(k+1)-h({\hat{\hat{x}}}_{l}^{i_{l}}(k+1|k+1),k+1)}$$

For the α(k+1)th iteration, if $\left|K_{\alpha (k+1)+1}^{*}(k+1)-K_{\alpha (k+1)}^{*}(k+1)\right|<\varepsilon_{K}$ ($\varepsilon_{K}$ is a given scalar), the final estimate output of the state at the time instant k+1 is given by

(16) $$\hat{\hat{x}}(k+1|k+1)={\hat{\hat{x}}}_{\alpha (k+1)}^{i_{l\alpha (k+1)}}(k+1|k+1)$$

**Figure 1:**
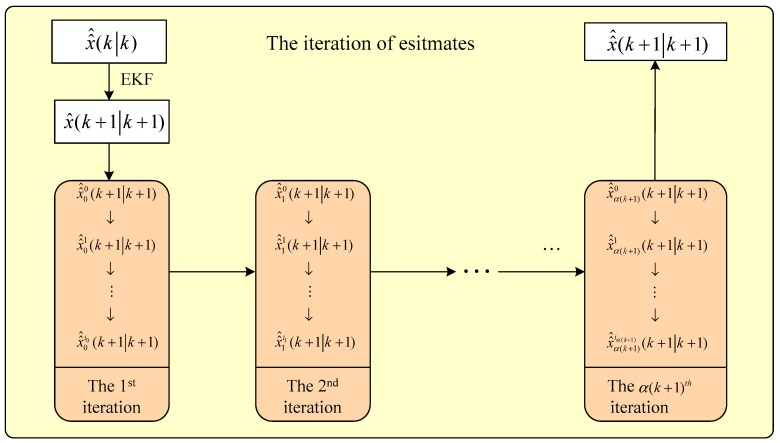
Flowchart of the iterative computational process of the estimates.

The flowchart of the iterative computational process of the estimates is illustrated in Figure 1.

### 3.4. Convergence of the Fixed-Point Filter

In order to ensure that the constructed fixed-point filter is convergent, it is necessary to conditionally constrain the fixed-point equation. In this subsection, the convergence of the fixed-point function (8) will be discussed.

Firstly, a necessary lemma is given as follows [26,27].

**Lemma 1.**  *An iterative function* φ(x) *has a unique fixed point* $x^{*}$ *in* [a,b] *, if*

(1) x∈[a,b]*, then*a≤φ(x)≤b;
(2) *There exists a positive scalar L, such that*$\left|\frac{\partial \varphi (x)}{\partial x}\right|\leq L$*, for*x∈[a,b].

Based on this lemma, the convergence conditions of the fixed-point function (8) are given as follows.

**Theorem 1.**  *For a given filter* $K^{*}(k+1)$ *, the fixed-point function (8) has a fixed point in [a,b], if the measurement function satisfies the following two conditions*

(1) $a+{\hat{\underline{y}}}_{\left[a,b\right]}\leq K^{*}(k+1)y(k+1)\leq b+{\bar{\hat{y}}}_{\left[a,b\right]}$ *, where*
  (17) $${\hat{\underline{y}}}_{\left[a,b\right]}=\min\left\{K^{*}(k+1)h(\hat{\hat{x}}(k+1|k+1),k+1)-\hat{\hat{x}}(k+1|k+1),where\;\hat{\hat{x}}(k+1|k+1)\in \left[a,b\right]\right\}$$
  (18) $${\bar{\hat{y}}}_{\left[a,b\right]}=\max\left\{K^{*}(k+1)h(\hat{\hat{x}}(k+1|k+1),k+1)-\hat{\hat{x}}(k+1|k+1),\;where\;\hat{\hat{x}}(k+1|k+1)\in \left[a,b\right]\right\}$$
(2) $\left|1-K^{*}(k+1)\frac{\partial h(\hat{\hat{x}}(k+1|k+1),k+1)}{\partial \hat{\hat{x}}(k+1|k+1)}\right|\leq L,\;\hat{\hat{x}}(k+1|k+1)\in \left[a,b\right]$.

**Proof.**  Denote

(19) $$\varphi (\hat{\hat{x}}(k+1|k+1))=\hat{\hat{x}}(k+1|k+1)+K^{*}(k+1)[y(k+1)-h(\hat{\hat{x}}(k+1|k+1),k+1)]$$

If $\hat{\hat{x}}(k+1|k+1)\in [a,b]$, according to Lemma 1, one has $a\leq \varphi (\hat{\hat{x}}(k+1|k+1))\leq b$. It means that

(20) $$a\leq \hat{\hat{x}}(k+1|k+1)+K^{*}(k+1)[y(k+1)-h(\hat{\hat{x}}(k+1|k+1),k+1)]\leq b$$

Then, the measurement function satisfies

(21) $$a\leq K^{*}(k+1)y(k+1)-{\hat{y}}_{\left[a,\;b\right]}\leq b$$

where

(22) $${\hat{y}}_{\left[a,\;b\right]}=K^{*}(k+1)h(\hat{\hat{x}}(k+1|k+1),k+1)-\hat{\hat{x}}(k+1|k+1),\;\hat{\hat{x}}(k+1|k+1)\in \left[a,b\right]$$

Using the upper bound in (17) and the lower bound in (18), one gets

(23) $$a+{\hat{\underline{y}}}_{\left[a,b\right]}\leq K^{*}(k+1)y(k+1)\leq b+{\bar{\hat{y}}}_{\left[a,b\right]}$$

The Jacobian value of $\varphi (\hat{\hat{x}}(k+1|k+1))$ is obtained by

(24) $$\frac{\partial \varphi (\hat{\hat{x}}(k+1|k+1))}{\partial \hat{\hat{x}}(k+1|k+1)}\;=\;1-K^{*}(k+1)\frac{\partial h(\hat{\hat{x}}(k+1|k+1),k+1)}{\partial \hat{\hat{x}}(k+1|k+1)}$$

Then, according to Lemma 1, the measurement function should satisfy

(25) $$\left|1-K^{*}(k+1)\frac{\partial h(\hat{\hat{x}}(k+1|k+1),k+1)}{\partial \hat{\hat{x}}(k+1|k+1)}\right|\leq L$$

Therefore, the fixed-point function (8) has a unique fixed point in [a,b], if the measurement function satisfies (23) and (25). The proof is completed. □

**Remark 3.**  *It is commonly known that not all fixed-point equations are convergent. The convergence conditions of the fixed-point function (8) are presented in Theorem 1, which is also the existing condition of the fixed-point iterative filter given in Section 3.3. If the convergence conditions in Theorem 1 are satisfied, the state estimate will be further updated by the fixed-point iterative filter shown in Section 3.3. Otherwise, the state estimate obtained by the EKF need not be updated and denoted as the final estimate at a certain time instant. The interval [a,b] can be set according to the actual demand, or by using the state prediction value with the 3-Delta rule. The norm could be 1-noum for the vector parameters.*

### 3.5. Steffensen’s Iterative Solution of the Fixed-Point Function

In Section 3.3, a nested iterative solution for the fixed-point filter was presented. In this section, Steffensen’s iterative method is used to accelerate the solution of the fixed-point function (8). Steffensen’s iterative method in the *l*th iteration is given as follows

(26) $${\hat{\hat{x}}}_{l}^{j+1}(k+1|k+1)={\hat{\hat{x}}}_{l}^{j}(k+1|k+1)-\frac{{\left(\phi ({\hat{\hat{x}}}_{l}^{j}(k+1|k+1))-{\hat{\hat{x}}}_{l}^{j}(k+1|k+1)\right)}^{2}}{\phi (\phi ({\hat{\hat{x}}}_{l}^{j}(k+1|k+1)))-2\phi ({\hat{\hat{x}}}_{l}^{j}(k+1|k+1))+{\hat{\hat{x}}}_{l}^{j}(k+1|k+1)},j=1,2,\cdots$$

Let $K_{0}^{*}(k+1)=\;K(k+1)$, then Steffensen’s iterative method can be given by

(27) $$\left\{ \begin{array}{c} {\hat{\hat{x}}}_{0}^{1}(k+1|k+1)={\hat{\hat{x}}}_{0}^{0}(k+1|k+1)-\frac{{\left(\phi ({\hat{\hat{x}}}_{0}^{0}(k+1|k+1))-{\hat{\hat{x}}}_{0}^{0}(k+1|k+1)\right)}^{2}}{\phi ((\phi ({\hat{\hat{x}}}_{0}^{0}(k+1|k+1)))-2\phi ({\hat{\hat{x}}}_{0}^{0}(k+1|k+1))+{\hat{\hat{x}}}_{0}^{0}(k+1|k+1)}\\ \vdots \\ {\hat{\hat{x}}}_{0}^{n_{0}-1}(k+1|k+1)={\hat{\hat{x}}}_{0}^{n_{0}-2}(k+1|k+1)-\frac{{\left(\phi ({\hat{\hat{x}}}_{0}^{n_{0}-2}(k+1|k+1))-{\hat{\hat{x}}}_{0}^{n_{0}-2}(k+1|k+1)\right)}^{2}}{\phi ((\phi ({\hat{\hat{x}}}_{0}^{n_{0}-2}(k+1|k+1)))-2\phi ({\hat{\hat{x}}}_{0}^{n_{0}-2}(k+1|k+1))+{\hat{\hat{x}}}_{0}^{n_{0}-2}(k+1|k+1)}\\ {\hat{\hat{x}}}_{0}^{n_{0}}(k+1|k+1)={\hat{\hat{x}}}_{0}^{n_{0}-1}(k+1|k+1)-\frac{{\left(\phi ({\hat{\hat{x}}}_{0}^{n_{0}-1}(k+1|k+1))-{\hat{\hat{x}}}_{0}^{n_{0}-1}(k+1|k+1)\right)}^{2}}{\phi ((\phi ({\hat{\hat{x}}}_{0}^{n_{0}-1}(k+1|k+1)))-2\phi ({\hat{\hat{x}}}_{0}^{n_{0}-1}(k+1|k+1))+{\hat{\hat{x}}}_{0}^{n_{0}-1}(k+1|k+1)} \end{array}\right.$$

where

(28) $$\phi ({\hat{\hat{x}}}_{0}^{i}(k+1|k+1))={\hat{\hat{x}}}_{0}^{i}(k+1|k+1)+K_{0}^{*}(k+1)(y(k+1)-h({\hat{\hat{x}}}_{0}^{i}(k+1|k+1))),i=0,1,\cdots ,n_{0}$$

(29) $$\phi ((\phi ({\hat{\hat{x}}}_{0}^{i}(k+1|k+1)))=\phi ({\hat{\hat{x}}}_{0}^{i}(k+1|k+1)+K_{0}^{*}(k+1)(y(k+1)-h((\phi ({\hat{\hat{x}}}_{0}^{i}(k+1|k+1)))),i=0,1,\cdots ,n_{0}$$

If $\left|{\hat{\hat{x}}}_{0}^{n_{0}}(k+1|k+1)-{\hat{\hat{x}}}_{0}^{n_{0}-1}(k+1|k+1)\right|<\varepsilon$, take ${\hat{\hat{x}}}_{0}^{n_{0}}(k+1|k+1)$ as the state estimate of the fixed-point filter with the filter gain $K_{0}^{*}(k+1)$.

Similar to (11), the filter gain can be updated by

(30) $$K^{*}{}_{1}(k+1)=\frac{{\hat{\hat{x}}}_{0}^{n_{0}}(k+1|k+1)-{\hat{\hat{x}}}_{0}^{n_{0}-1}(k+1|k+1)}{y(k+1)-h({\hat{\hat{x}}}_{0}^{n_{0}}(k+1|k+1),k+1)}$$

Substituting the new filter gain above, the fixed-point function is represented as

(31) $$\hat{\hat{x}}(k+1|k+1)=\hat{\hat{x}}(k+1|k+1)+K^{*}{}_{1}(k+1)[y(k+1)-h(\hat{\hat{x}}(k+1|k+1),k+1)-E\{v(k+1)\}]$$

According to Steffensen’s iterative method, one has

(32) $$\left\{ \begin{array}{c} {\hat{\hat{x}}}_{1}^{1}(k+1|k+1)={\hat{\hat{x}}}_{1}^{0}(k+1|k+1)-\frac{{\left(\phi ({\hat{\hat{x}}}_{1}^{0}(k+1|k+1))-{\hat{\hat{x}}}_{1}^{0}(k+1|k+1)\right)}^{2}}{\phi ((\phi ({\hat{\hat{x}}}_{1}^{0}(k+1|k+1)))-2\phi ({\hat{\hat{x}}}_{1}^{0}(k+1|k+1))+{\hat{\hat{x}}}_{1}^{0}(k+1|k+1)}\\ \vdots \\ {\hat{\hat{x}}}_{1}^{n_{1}-1}(k+1|k+1)={\hat{\hat{x}}}_{1}^{n_{1}-2}(k+1|k+1)-\frac{{\left(\phi ({\hat{\hat{x}}}_{1}^{n_{1}-2}(k+1|k+1))-{\hat{\hat{x}}}_{1}^{n_{1}-2}(k+1|k+1)\right)}^{2}}{\phi ((\phi ({\hat{\hat{x}}}_{1}^{n_{1}-2}(k+1|k+1)))-2\phi ({\hat{\hat{x}}}_{1}^{n_{1}-2}(k+1|k+1))+{\hat{\hat{x}}}_{1}^{n_{1}-2}(k+1|k+1)}\\ {\hat{\hat{x}}}_{1}^{n_{1}}(k+1|k+1)={\hat{\hat{x}}}_{1}^{n_{1}-1}(k+1|k+1)-\frac{{\left(\phi ({\hat{\hat{x}}}_{1}^{n_{1}-1}(k+1|k+1))-{\hat{\hat{x}}}_{1}^{n_{1}-1}(k+1|k+1)\right)}^{2}}{\phi ((\phi ({\hat{\hat{x}}}_{1}^{n_{1}-1}(k+1|k+1)))-2\phi ({\hat{\hat{x}}}_{1}^{n_{1}-1}(k+1|k+1))+{\hat{\hat{x}}}_{1}^{n_{1}-1}(k+1|k+1)} \end{array}\right.$$

where

(33) $$\phi ({\hat{\hat{x}}}_{1}^{i}(k+1|k+1))={\hat{\hat{x}}}_{1}^{i}(k+1|k+1)+K_{1}^{*}(k+1)(y(k+1)-h({\hat{\hat{x}}}_{1}^{i}(k+1|k+1))),i=0,1,\cdots ,n_{1}$$

(34) $$\phi ((\phi ({\hat{\hat{x}}}_{1}^{i}(k+1|k+1)))=\phi ({\hat{\hat{x}}}_{1}^{i}(k+1|k+1)+K_{1}^{*}(k+1)(y(k+1)-h((\phi ({\hat{\hat{x}}}_{1}^{i}(k+1|k+1)))),i=0,1,\cdots n_{1}$$

If $\left|{\hat{\hat{x}}}_{1}^{n_{1}}(k+1|k+1)-{\hat{\hat{x}}}_{1}^{n_{1}-1}(k+1|k+1)\right|<\varepsilon$, take ${\hat{\hat{x}}}_{1}^{n_{1}}(k+1|k+1)$ as the state estimate of the fixed-point filter with the filter gain $K^{*}{}_{1}(k+1)$.

Similarly, the filter gain can be updated by

(35) $$K_{j}^{*}(k+1)=\frac{{\hat{\hat{x}}}_{j-1}^{n_{j-1}}(k+1|k+1)-{\hat{\hat{x}}}_{j-1}^{n_{j-1}-1}(k+1|k+1)}{y(k+1)-h({\hat{\hat{x}}}_{j-1}^{n_{j-1}}(k+1|k+1),k+1)}$$

The fixed-point filter is constructed as

(36) $$\hat{\hat{x}}(k+1|k+1)=\hat{\hat{x}}(k+1|k+1)+K_{j}^{*}(k+1)[y(k+1)-h(\hat{\hat{x}}(k+1|k+1),k+1)-E\{v(k+1)\}]$$

Steffensen’s iterative solution of the fixed-point filter above is obtained by

(37) $$\left\{ \begin{array}{c} {\hat{\hat{x}}}_{j}^{1}(k+1|k+1)={\hat{\hat{x}}}_{j}^{0}(k+1|k+1)-\frac{{\left(\phi ({\hat{\hat{x}}}_{j}^{0}(k+1|k+1))-{\hat{\hat{x}}}_{j}^{0}(k+1|k+1)\right)}^{2}}{\phi ((\phi ({\hat{\hat{x}}}_{j}^{0}(k+1|k+1)))-2\phi ({\hat{\hat{x}}}_{j}^{0}(k+1|k+1))+{\hat{\hat{x}}}_{j}^{0}(k+1|k+1)}\\ \vdots \\ {\hat{\hat{x}}}_{j}^{n_{j}-1}(k+1|k+1)={\hat{\hat{x}}}_{j}^{n_{j}-2}(k+1|k+1)-\frac{{\left(\phi ({\hat{\hat{x}}}_{j}^{n_{j}-2}(k+1|k+1))-{\hat{\hat{x}}}_{j}^{n_{j}-2}(k+1|k+1)\right)}^{2}}{\phi ((\phi ({\hat{\hat{x}}}_{j}^{n_{j}-2}(k+1|k+1)))-2\phi ({\hat{\hat{x}}}_{j}^{n_{j}-2}(k+1|k+1))+{\hat{\hat{x}}}_{j}^{n_{j}-2}(k+1|k+1)}\\ {\hat{\hat{x}}}_{j}^{n_{j}}(k+1|k+1)={\hat{\hat{x}}}_{j}^{n_{j}-1}(k+1|k+1)-\frac{{\left(\phi ({\hat{\hat{x}}}_{j}^{n_{j}-1}(k+1|k+1))-{\hat{\hat{x}}}_{j}^{n_{j}-1}(k+1|k+1)\right)}^{2}}{\phi ((\phi ({\hat{\hat{x}}}_{j}^{n_{j}-1}(k+1|k+1)))-2\phi ({\hat{\hat{x}}}_{j}^{n_{j}-1}(k+1|k+1))+{\hat{\hat{x}}}_{j}^{n_{j}-1}(k+1|k+1)} \end{array}\right.$$

where

(38) $$\phi ({\hat{\hat{x}}}_{j}^{i}(k+1|k+1))={\hat{\hat{x}}}_{j}^{i}(k+1|k+1)+K_{j}^{*}(k+1)(y(k+1)-h({\hat{\hat{x}}}_{j}^{i}(k+1|k+1))),i=0,1,\cdots ,n_{j}$$

(39) $$\phi ((\phi ({\hat{\hat{x}}}_{j}^{i}(k+1|k+1)))=\phi ({\hat{\hat{x}}}_{j}^{i}(k+1|k+1)+K_{j}^{*}(k+1)(y(k+1)-h((\phi ({\hat{\hat{x}}}_{j}^{i}(k+1|k+1)))),i=0,1,\cdots ,n_{j}$$

If $\left|{\hat{\hat{x}}}_{j}^{n_{j}}(k+1|k+1)-{\hat{\hat{x}}}_{j}^{n_{j}-1}(k+1|k+1)\right|<\varepsilon$, take ${\hat{\hat{x}}}_{j}^{n_{j}}(k+1|k+1)$ as the solution of the fixed-point filter with the filter gain $K_{j}^{*}(k+1)$.

If $\left|K_{t}^{*}(k+1)-K_{t-1}^{*}(k+1)\right|<\varepsilon_{K}$, the state estimate ${\hat{\hat{x}}}_{t}^{n_{t}}(k+1|k+1)$ obtained by the fixed-point iteration process with the filter gain $K_{t}^{*}(k+1)$ is the final solution of the fixed-point filter.

**Remark 4.**  *Compared with the nested iterative solution method in Section 3.3, Steffensen’s iterative method can accelerate the solution process of the fixed-point function (8). Because of this, the filtering result of the two fixed-point filters may be slightly different.*

**Remark 5.**  *It should be noted that the conditions for judging whether the iterative process ends in the solution methods of the fixed-point function are to compare the differences between the results of two adjacent iterations. However, the final purposes of the updating methods are reducing the differences between the estimates and the active values of the system states. It is a pity that the active values of the system states are unavailable. Therefore, the difference between the active value and the prediction values of the measurement by different methods are compared in the simulation to determine whether the updating is necessary.*

## 4. Simulation

In this section, two comparison simulation examples are provided to illustrate the feasibility and the effectiveness of the proposed EKF-based fixed-point iterative filters (the one provided in Section 3.3 is denoted as FEKF, while the one provided in Section 3.5 is denoted SFEKF), which are compared with the classical EKF and UKF methods. 

**Simulation I**. Considering the following non-linear system

(40) $$\left\{ \begin{array}{l} x(k+1)=0.5x(k)+2.5x(k)/(1+x^{2}(k))+w(k+1,k)\\ y(k+1)=x^{2}(k+1)+v(k+1)+0.2\cos(k/\pi ) \end{array}\right.$$

where w(k+1,k), v(k+1) are respectively the process noise and the measurement noise, which satisfy the zero-mean Gaussian distributions with the variances Q=0.1, R=0.001. The initial state estimation and its error variance are respectively x(0)=2, p(0)=0.01.

In order to reduce the effects of random noise on the comparison results, a Monte Carlo simulation was repeated 50 times. The simulation results were as follows. 

**Figure 2:**
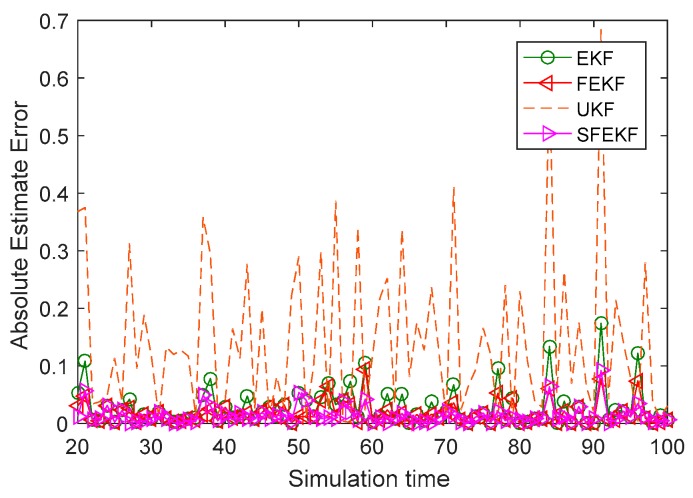
The absolute error curves of the four methods.

As shown in Figure 2, compared with the absolute error curves of the classical EKF and UKF, the error curves of the proposed iterative fixed-point filters were lower at almost all 80 simulation time instants. The main cause of the higher absolute error curve of the EKF was the discarded higher order terms of the Taylor series in the linearization processes. Since the proposed fixed-point filters iteratively updated the estimates of the EKF, the absolute error curve was lower than that of the EKF. The UKF approximated the statistics of the state estimates by using unscented transformation, and the estimate accuracy of UKF was related to the filter parameters. The estimate accuracy of the UKF was the lowest, although several possible filter parameters were tested. The mean weightings $W_{i}^{m}$ and variance weightings $W_{i}^{c}$ of the UKF were respectively set as $W_{i}^{m}\;=\;\left\{ \begin{array}{l} \lambda /(n+\lambda ),\;i=1\\ 1/(2n+2\lambda ),\;i=2,3 \end{array}\right.$, $W_{i}^{c}\;=\;\left\{ \begin{array}{l} \lambda /(n+\lambda )+(1-\alpha^{2}+\beta ),\;i=1\\ 1/(2+2\lambda ),\;i=2,3 \end{array}\right.$, where $\lambda \;=\alpha^{2}(n+\kappa )-n,\;\alpha =1,\;\beta =2,\;\kappa =3-n,\;n=1$. 

The fixed-point iterative filter based on the extended Kalman filter can compensate the influence of discarded higher order terms of the Taylor series. As a result, the filtering results of the methods proposed in this paper were better than others. The mean values of the absolute error of the four comparison methods in Table 1 illustrated the feasibility and effectiveness of the proposed methods. In the same simulation conditions, the mean absolute error of the FEKF was 0.0178 and the mean absolute error of the SFEKF was 0.0141, while the others were 0.0278 for the EKF, and 0.1426 for the UKF, respectively. As shown in Table 2, the computation complexity of the EKF was the smallest, and that of the UKF was the largest, due to the computation of the sigma points. The FEKF and SFEKF were designed to update the estimation results of the EKF, and accordingly, their computation complexities were larger than that of the EKF. Steffensen’s iterative method was used to accelerate the solution of the fixed-point function (8), therefore, the CPU time cost of repeating Monte Carlo simulations 50 times was smaller with the SFEKF than with the FEKF.

**Figure 3:**
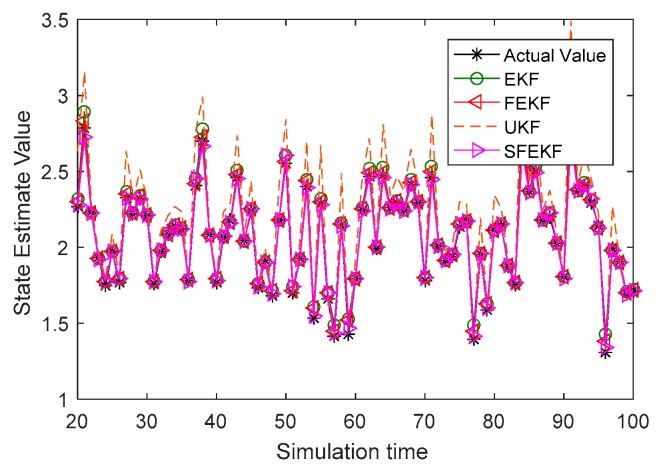
The estimate curves of the four methods and the actual value curve.

**Figure 4:**
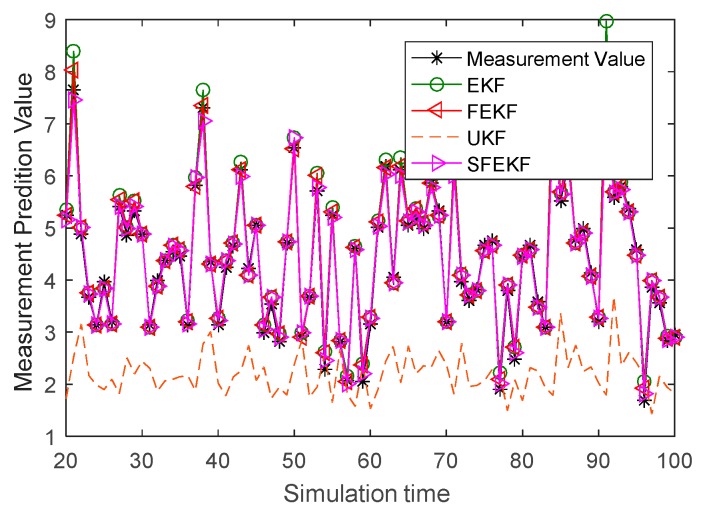
The measurement prediction curves of the four methods and the actual observation curve.

It is noted that the estimates obtained by the proposed methods were the closest to the actual values, as shown in Figure 3. Due to the influence of the discarded higher order items of the Taylor series system functions, the estimate accuracy of the EKF method was relatively poor. The influence was reduced using a fixed-point iterative filter. The measurement prediction results in Figure 4 also illustrated the feasibility and the effectiveness of the proposed fixed-point iterative filters. 

**Simulation II.** Consider the following target tracking system, in which the target is in nearly constant velocity:

(41) $$\left[\begin{array}{c} x(k+1)\\ \dot{x}(k+1)\\ y(k+1)\\ \dot{y}(k+1) \end{array}\right]=\left[\begin{array}{cccc} 1 & T & 0 & 0\\ 0 & 1 & 0 & 0\\ 0 & 0 & 1 & T\\ 0 & 0 & 0 & 1 \end{array}\right]\left[\begin{array}{c} x(k)\\ \dot{x}(k)\\ y(k)\\ \dot{y}(k) \end{array}\right]+\left[\begin{array}{cc} T^{2}/2 & 0\\ T & 0\\ 0 & T^{2}/2\\ 0 & T \end{array}\right]\left[\begin{array}{l} w_{x}(k+1,k)\\ w_{y}(k+1,k) \end{array}\right]$$

The target was initially located at (0, 1400), with an initial velocity of (1.8, −9.5). A radar was located at the origin of the polar coordinate. The measurement equation was given by

(42) $$\left\{ \begin{array}{l} \theta (k)=\arctan\left(\frac{y(k)}{x(k)}\right)+v_{\theta }(k)\\ r(k)=\sqrt{{\left(x(k)\right)}^{2}+{\left(y(k)\right)}^{2}}+v_{r}(k) \end{array}\right.$$

**Figure 5:**
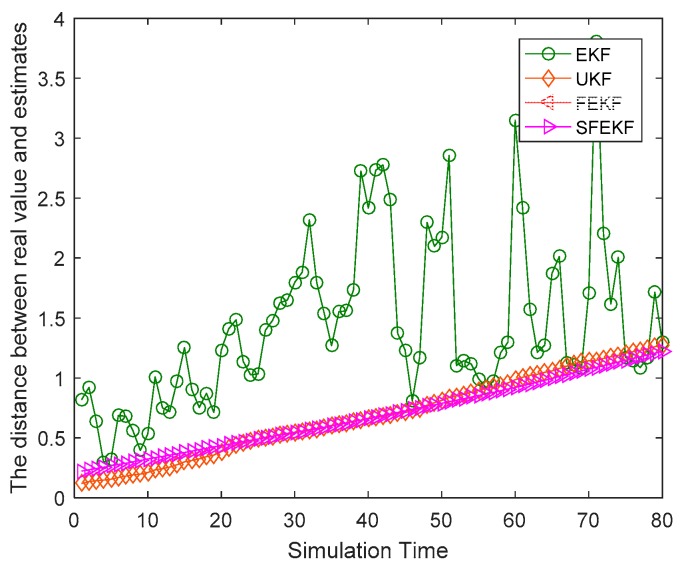
The distance between the real value and estimates for the four methods.

**Figure 6:**
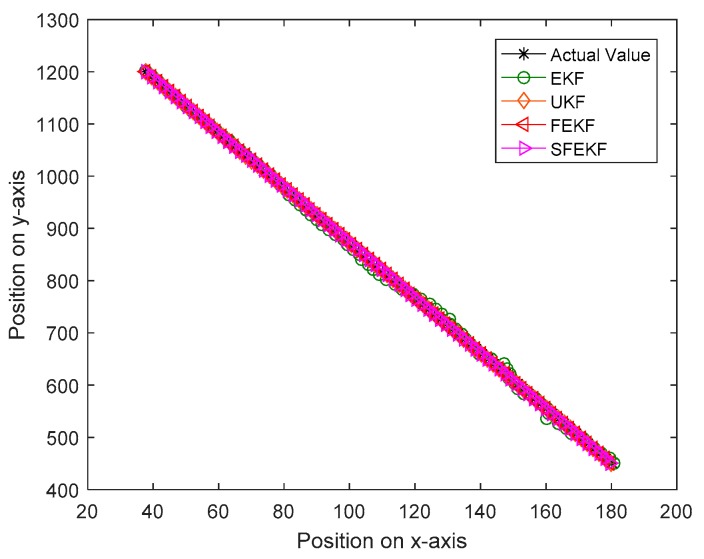
The real position and estimates for the four methods.

The variances of $\left[\begin{array}{l} w_{x}(k+1,k)\\ w_{y}(k+1,k) \end{array}\right]$ and $\left[\begin{array}{l} v_{\theta }(k)\\ v_{r}(k) \end{array}\right]$ were $\left[\begin{array}{cc} 10^{-4} & 0\\ 0 & 10^{-4} \end{array}\right]$ and $\left[\begin{array}{cc} 10^{-2} & 0\\ 0 & 10 \end{array}\right]$. The sampling period was 1. The simulation results are shown in Figure 5 and Figure 6.

As shown in Figure 6, the proposed FEKF and SFEKF methods effectively tracked the target with the radar measurements. The two proposed methods obtained more accurate estimates of the target than the EKF for the updating method with the fixed-point theory, as shown in Figure 5 and Table 3. 

## 5. Conclusions

As a development of the classic extended Kalman filter, the fixed-point iterative updating method was studied in this paper, drawing on fixed-point theory. On the basis of the extended Kalman filter, a fixed-point function was provided to update the state estimate obtained by the EKF and reduce the influence of the discarded higher order items of the Taylor series. The fixed-point function was solved by the nested iterative method and Steffensen’s iterative method and resulted in two fixed-point iterative filters. The convergence conditions of the fixed-point function were also studied, as were the existing conditions of fixed-point iterative filters.

## Figures and Tables

**Table 1:** The mean absolute errors (MAEs) of four comparison methods.

Methods	EKF	UKF	FEKF	SFEKF
MAEs	0.0278	0.1426	0.0178	0.0141

**Table 2:** The CPU time cost of 50 Monte Carlo simulations for the four comparison methods.

Methods	EKF	UKF	FEKF	SFEKF
CPU time	0.1406	2.4063	2.2656	0.3594

**Table 3:** The mean distances between the real value and estimates for the four comparison methods.

Methods	EKF	UKF	FEKF	SFEKF
Mean Distance	1.1913	0.5573	0.5530	0.5530
